# Electrodermal Activity Is Sensitive to Cognitive Stress under Water

**DOI:** 10.3389/fphys.2017.01128

**Published:** 2018-01-17

**Authors:** Hugo F. Posada-Quintero, John P. Florian, Alvaro D. Orjuela-Cañón, Ki H. Chon

**Affiliations:** ^1^Department of Biomedical Engineering, University of Connecticut, Storrs, CT, United States; ^2^Navy Experimental Diving Unit, Panama City, FL, United States; ^3^Faculty of Electronics and Biomedical Engineering, Universidad Antonio Nariño, Bogotá, Colombia

**Keywords:** sympathetic function, electrodermal activity, water immersion, autonomic nervous system, power spectral density, stroop test

## Abstract

When divers are at depth in water, the high pressure and low temperature alone can cause severe stress, challenging the human physiological control systems. The addition of cognitive stress, for example during a military mission, exacerbates the challenge. In these conditions, humans are more susceptible to autonomic imbalance. Reliable tools for the assessment of the autonomic nervous system (ANS) could be used as indicators of the relative degree of stress a diver is experiencing, which could reveal heightened risk during a mission. Electrodermal activity (EDA), a measure of the changes in conductance at the skin surface due to sweat production, is considered a promising alternative for the non-invasive assessment of sympathetic control of the ANS. EDA is sensitive to stress of many kinds. Therefore, as a first step, we tested the sensitivity of EDA, in the time and frequency domains, specifically to *cognitive* stress during water immersion of the subject (albeit with their measurement finger dry for safety). The data from 14 volunteer subjects were used from the experiment. After a 4-min adjustment and baseline period after being immersed in water, subjects underwent the Stroop task, which is known to induce cognitive stress. The time-domain indices of EDA, skin conductance level (SCL) and non-specific skin conductance responses (NS.SCRs), did not change during cognitive stress, compared to baseline measurements. Frequency-domain indices of EDA, EDASymp (based on power spectral analysis) and TVSymp (based on time-frequency analysis), did significantly change during cognitive stress. This leads to the conclusion that EDA, assessed by spectral analysis, is sensitive to cognitive stress in water-immersed subjects, and can potentially be used to detect cognitive stress in divers.

## Introduction

Electrodermal activity (EDA) dynamics exhibit both tonic and phasic changes, regulated by sympathetic innervation of the sweat glands. Variations in EDA are a product of the innervation of sweat glands that results in changing levels of sweat in the ducts (Edelberg, 1993). Functionally, EDA is associated with central mechanisms that play different roles, including gross movements, thermoregulatory sweating, affective processes, orientation and attention, and fine control (Edelberg, 1973; Hugdahl, 2001; Boucsein, 2016).

New non-invasive instrumentation for sympathetic nervous system dynamics using EDA has gained some popularity in recent years (Colbert et al., 2011; Boucsein et al., 2012; Freeman and Chapleau, 2013). EDA is a measure of the changes in electrical conductance of the skin, with strong correlation to sweat production. Because there is no parasympathetic innervation of eccrine sweat glands, EDA reflects only activity within the sympathetic branch of the autonomic nervous system (ANS). As such, EDA measures have been used recently to assess the sympathetic nervous system arousal (Critchley, 2002).

Time-domain analysis of EDA decomposes the signal into two quantitative measures: skin conductance level (SCL) and skin conductance responses (SCRs) (Boucsein et al., 2012). SCL (usually expressed in microsiemens, μS) is a measure related to the slow tonic shifts of EDA. SCL is typically computed as a mean of several measurements taken during a specific non-stimulation rest period. Skin conductance responses (SCRs) are the rapid phasic transient events contained in the EDA signal. The non-specific SCRs (NS.SCRs) are the number of SCRs in a period of time, and are a measure of tonic stress produced during a sustained stimuli. NS.SCRs are regularly expressed as the number of responses per minute (Boucsein et al., 2012). Recently, frequency-domain analyses of EDA in response to stress have been conducted (Posada-Quintero et al., 2016a,b). The time-invariant (EDASymp, based on power spectral analysis) and time-variant (TVSymp, based on time-frequency analysis) indices have shown to be sensitive to cognitive, physical and orthostatic stress.

Elevated hyperbaric pressure, nitrogen loading, hyperoxia, and cold temperatures often associated with increasing depth underwater are known to cause severe challenges to human physiological control systems (Bosco et al., 2007). During military dives, however, there are usually also intense cognitive challenges. These combined conditions may result in neurological and cardiovascular problems, conditions linked to autonomic imbalance (Kurita, 2002; Hirayanagi et al., 2003; Bai et al., 2009; Hansel et al., 2009; Florian, 2010). Means for early detection of affected dynamics of the ANS in underwater conditions are needed. As discussed, EDA has shown to be a promising alternative to measures such as heart rate variability for the non-invasive assessment of the sympathetic tone. All aforementioned studies were carried out in controlled, dry conditions. It is not clear whether EDA would be functional and show diagnostic characteristics of any type of stress for an immersed subject. To this end, the aim of this work was to examine the responsiveness of EDA to only cognitive stress, as a first step, for subjects immersed in water. An understanding even of the degree of cognitive stress that a diver is under, during a mission, is still useful.

Changes in EDA can be induced by startle (instantaneous) or tonic stimuli (Boucsein et al., 2012). To assess the responsiveness of EDA underwater, in this study we selected the widely-used and standardized stimulus (Stroop task) that induces cognitive stress on humans, because it is one of the true and tried stimuli to excite the subjects' skin sympathetic system.

There are two categories of methods to analyze EDA: time domain (also referred as structural analysis), and frequency domain. The time-domain measures give a lot of details and information, but the detection of the key points (i.e., the onset, peak, and offset of the measured SCRs) is not easy to automate due to frequent occurrence of confounding factors (Boucsein et al., 2012; Braithwaite et al., 2013; Taamneh et al., 2017), although progress has been made in this respect (Benedek and Kaernbach, 2010; Bach and Friston, 2013; Chaspari et al., 2015; Greco et al., 2015; Tsiamyrtzis et al., 2016). The spectral analysis of EDA provides less detail, but are on average more robust, easier to implement, and has previously shown good sensitivity of EDA to tonic cognitive stress. We have used both approaches (time and frequency domain) for this study.

In this study, we have not recreated the extreme situations a diver can encounter. Our aim was to assess the responsiveness of EDA to cognitive stress under mild environmental alterations, like the ones produced by immersion in a one-person sized pool. To our best knowledge this is the first study examining the feasibility of using EDA to assess cognitive stress during water immersion.

## Materials and methods

### Protocol

Eighteen healthy volunteers (14 males, 4 females) of ages ranging from 19 to 54 years old (27 ± 10; mean ± SD), weight 66 ± 6 kg, and height 172 ± 8 cm, were enrolled in this study. Participants were asked to avoid caffeine and alcohol for 24 h preceding the test, and instructed to fast for at least 3 h before testing. The experiments were carried out in a quiet room. This study was carried out in accordance with the recommendations of the Institutional Review Board of The University of Connecticut, with written informed consent from all subjects. All subjects gave written informed consent in accordance with the Declaration of Helsinki. The study protocol was approved by the Institutional Review Board of The University of Connecticut. The galvanic skin response amplifier FE116 (fully isolated AC excitation and automatic zeroing low voltage amplifier, 22 mV_rms_ @75 Hz, ADINSTRUMENTS) was used to collect EDA during the test. No on-line filtering was applied during the signal recording. EDA electrodes were placed on the index and middle fingers for all subjects. Skin was prepared with alcohol before placing the electrodes. Signals were digitized using a PowerLab system at 100 Hz, with 12 bits resolution.

**Figure 1:**
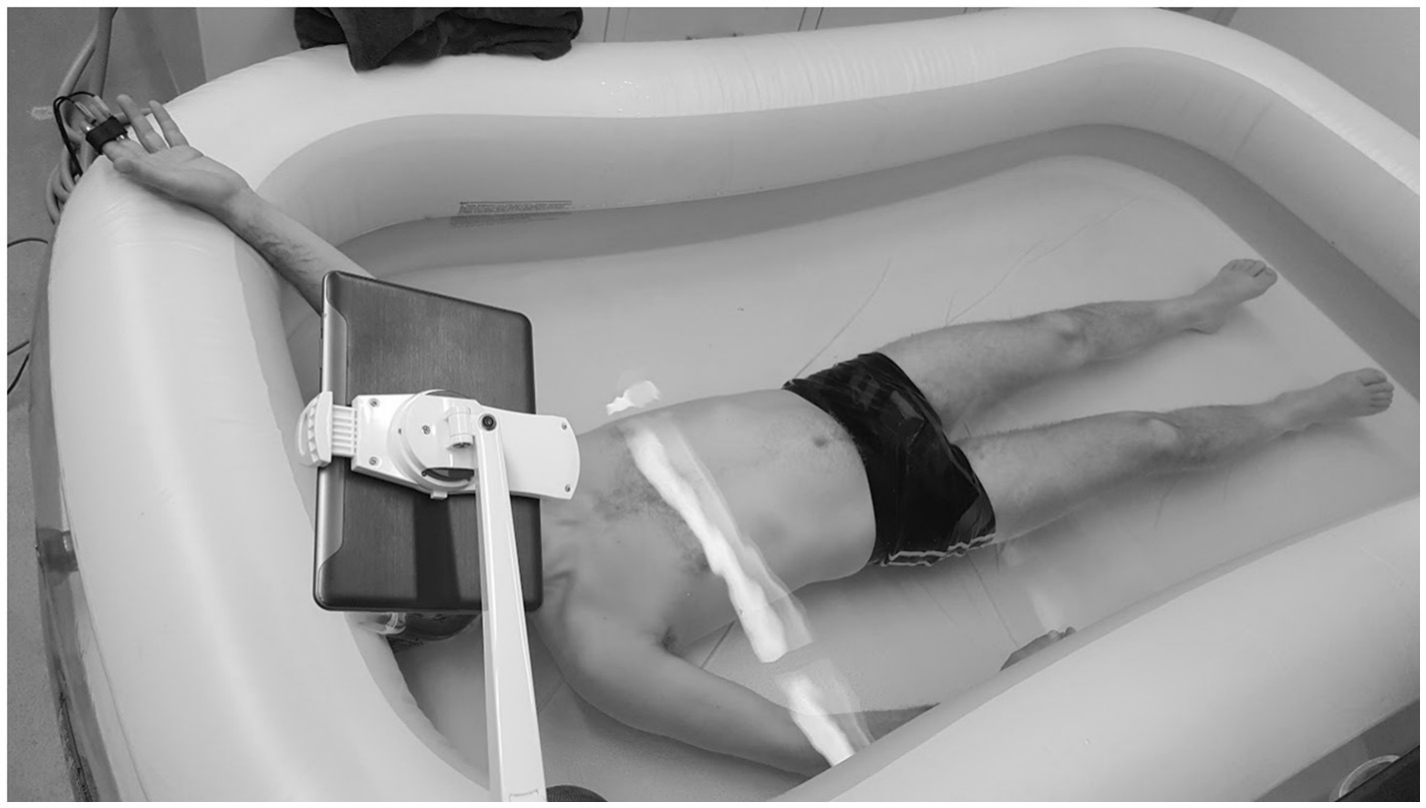
Experimental setup. EDA data was collected in the middle and index fingers on left hand, while subjects underwent Stroop test underwater. Written informed consent was obtained from the individual for the publication of this image. Values are expressed as mean ± standard deviation.

Figure 1 illustrates the experimental setup. Subjects were required to lie inside a 150 gallon inflatable pool. The temperature of the water was regulated with the subject to assure comfort. During the test, subjects were immersed in water wearing goggles, breathing through a snorkel. Since a small current was applied to one of the electrodes, the left (instrumented) hand remained out of the water for EDA measurements. Before the test started, the subjects relaxed for 4 min, so that they could get used to breathing through the snorkel under water. For the test, 2 min of baseline were recorded with subjects relaxing in the supine position under water. Then, subjects were asked to perform the Stroop task, in which the subject was shown congruent visualizations (the word was written in the color it expressed) and incongruent visualizations (the word and the color it was printed in were different). Subjects were asked to determine the color of a word which named a color, to induce cognitive stress (Stroop, 1935). The words and colors were “blue,” “yellow,” “green,” “red,” “purple,” and “black.” The background also changed to be randomly congruently or incongruently colored with the word. A tablet-PC version of the original Stroop task was developed in our lab using customized software, and during the Stroop task the tablet was put in front of subjects' faces using a stand. The Stroop task was 2 min in duration. Subjects practiced the Stroop task saying the color out loud, before going underwater. Then, subjects performed the task of determining the color mentally, as the snorkel impeded the subjects' ability to speak.

### Signal processing

Indices of EDA were computed in the time and frequency domains. In the time domain, the EDA signal was decomposed into tonic and phasic components, using the convex optimization approach (Greco et al., 2015). The SCL [μS], was computed as the mean value of the tonic component of EDA taken during the 2-min period (Boucsein et al., 2012). The NS.SCRs index was computed as the number of SCRs during the 2-min period.

For frequency-domain analysis, EDA signals were down–sampled to 2 Hz. Before down-sampling, the data were filtered with an 8th-order Chebyshev Type I low-pass filter (0.8 Hz). Down-sampling from 100 Hz to 2 Hz was performed in two steps (using consecutive down-sampling factors of 1/20 and 1/10, respectively). Finally, signals were high-pass filtered (0.01 Hz, Butterworth, 8th order) to remove any trend.

For time-invariant spectral analysis, the power spectra of EDA signals were calculated using Welch's periodogram with 50% data overlap. A Blackman window (length of 128 points) was applied to each segment, the Fast Fourier Transform was calculated for each windowed segment, and the power spectra of the segments were averaged. The dynamics of the EDA spectrum are largely confined to frequencies less than 0.4 Hz as observed in this work and as reported (Shimomura et al., 2008; Posada-Quintero et al., 2016a). Based on the spectrum of EDA, EDASympn [n.u.] was computed as the normalized power of EDA in the range 0.045 to 0.25 Hz.

For time-varying analysis of EDA, the time-frequency representation (TFR) of EDA was computed using variable frequency complex demodulation (VFCDM), a time-frequency spectral (TFS) analysis technique that provides accurate amplitude estimates and one of the highest time-frequency resolutions (Chon et al., 2009). The components comprising the frequency power in the range from 0.08 to 0.24 Hz were used to compute the time-varying index of EDA, TVSymp (Posada-Quintero et al., 2016b).

### Statistics

The measures of EDA collected underwater were SCL, NS.SCRs, EDASympn and TVSymp. The normality of the indices was tested using the one-sample Kolmogorov-Smirnov test (Massey Jr, 1951; Miller, 1956; Wang et al., 2003). All indices met normality criteria. The paired *t*-test was applied to test the null hypothesis that the above-mentioned indices during the Stroop test are equal to the baseline values.

## Results

Four subjects (3 male, 1 female) reported a high level of stress induced by the mere circumstance of being submerged in water. Data from these subjects were excluded from analysis owing either to the subjects not being able to finish the test, or their having reported that they could not properly execute the Stroop task under water. Although these latter subjects exhibited responsive EDA under water, they were excluded from further analysis as no difference between baseline and Stroop task could be observed. For these latter subjects, their EDA level varied highly both during baseline and during the Stroop task stages. Also, SCRs were observed with high frequency during baseline and Stroop task. Besides this small group of subjects exhibiting a big stress reaction to water immersion, the other subjects were comfortable with snorkel breathing while performing Stroop tasks. Thus, the data set analyzed consists of 14 subjects (11 males, 3 females).

**Figure 2:**
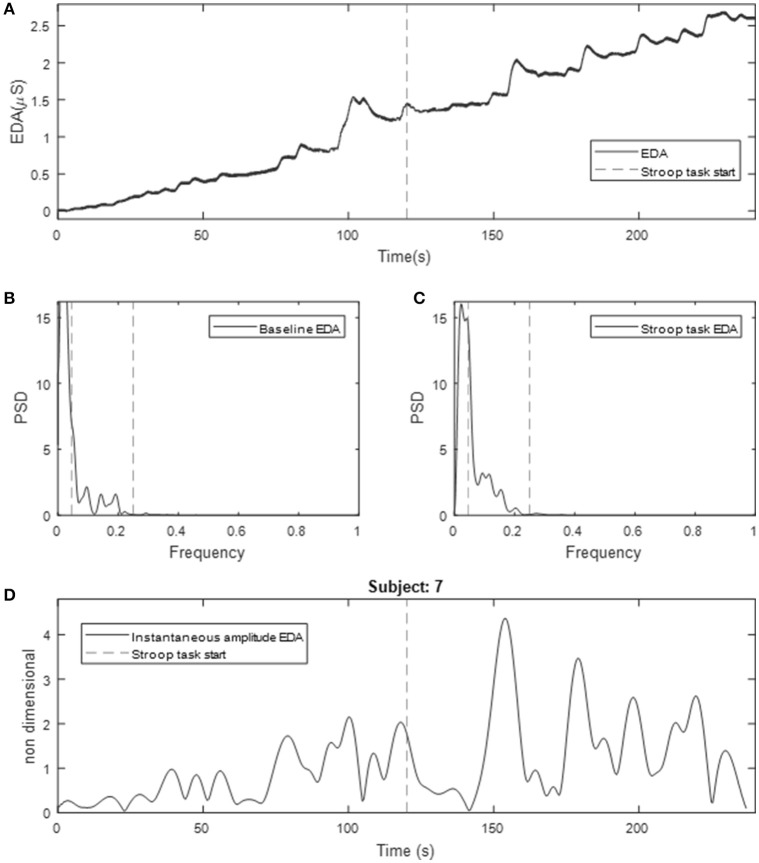
Obtained EDA for a given subject undergoing Stroop test underwater. **(A)** Raw EDA data, **(B)** spectrum of baseline measurement; **(C)** spectrum of Stroop test measurement (dotted lines represent 0.05 Hz and 0.25 Hz); **(D)** instantaneous amplitude computed using time-varying spectral analysis (line represents the time when Stroop task starts). Values are expressed as mean ± standard deviation.

Figure 2 (top) shows the resulting EDA data for 2 min of baseline and Stroop task for a given subject. Note the tonic increment in the level of EDA. Interestingly, almost all subjects exhibited such behavior, not only during the Stroop test, but also during the baseline measurements when no stimulus was presented to the subject. Note that during Stroop test the frequency of NS.SCRs increased for this subject. The power spectra of the EDA signal for a given subject during baseline and Stroop test are included in Figure 2 (middle). Note how this subject exhibited marked differences between baseline and Stroop test. The spectral power of EDA beyond 0.25 Hz is minimal.

Previously, we found that more than 95% of the spectral power of EDA is in the range 0 to 0.25 Hz, for subjects undergoing cognitive and other types of stress in supine position, in dry conditions (Posada-Quintero et al., 2016a). In this study, we have computed the percentage of time-invariant spectral power of EDA within frequency bands of 0.05 Hz, for immersed subjects (Table 1). The first range, 0 to 0.05 Hz, comprises most of the power for baseline and Stroop task measurements, and its percentage power is reduced from baseline to Stroop task. The bands covering 0.05 to 0.25 Hz were increased during Stroop task, compared to baseline. About 95% of the power is comprised in the range 0 to 0.25 Hz. Similarly, the percentage of the power of components of the time-varying spectra of EDA are presented in Table 2. The first component, centered on 0.04 Hz, comprises most of the frequency of EDA. Significant differences were found for the abovementioned first component, as well as second (around 0.12 Hz) and third (around 0.2 Hz) components, with the power reduced for the first and increased for the latter two. Similar results were achieved in a previous study, where EDA data were analyzed in time-frequency domain for subjects undergoing several types of stress (Posada-Quintero et al., 2016b).

**Table 1:** Percentage of time-invariant spectral power within the frequency bands of EDA for immersed subjects during baseline and Stroop task stages.

**Range (Hz)**	**Baseline (%)**	**Stroop task (%)**
0 to 0.05	67.8 ± 17.8	59.4 ± 22.6
0.05 to 0.1	14.6 ± 9.25	17.8 ± 9.32
0.1 to 0.15	5.98 ± 4.51	8.56 ± 6.07
0.15 to 0.2	3.53 ± 3.86	5.84 ± 6.74^*^
0.2 to 0.25	1.81 ± 1.54	2.93 ± 2.74
0.25 to 0.3	1.61 ± 1.85	1.51 ± 1.19
0.3 to 0.35	1.89 ± 4	1.44 ± 2.07
0.35 to 0.4	1.15 ± 1.77	1.02 ± 1.46
> 0.4	1.54 ± 1.62	1.5 ± 1.8

* *Statistically significantly higher with respect to baseline (p < 0.05)*.

**Table 2:** Percentage of power within the time-varying spectral components of EDA for immersed subjects.

**Component *fo* (Hz)**	**Baseline**	**Stroop task**
0.04	84.4 ± 12.4	69.7 ± 20.1^*^
0.12	6.22 ± 5.03	11.7 ± 6.56^*^
0.2	2.8 ± 3.45	6.34 ± 6.88^*^
0.28	1.27 ± 1.26	2.12 ± 2.38
0.36	0.861 ± 1.03	1.38 ± 1.99
0.44	0.283 ± 0.307	0.496 ± 0.644
0.52	0.148 ± 0.113	0.284 ± 0.462
0.6	0.115 ± 0.118	0.138 ± 0.133
0.68	0.0762 ± 0.0785	0.102 ± 0.075
0.76	0.0584 ± 0.0836	0.0702 ± 0.0904
0.84	0.0475 ± 0.112	0.0275 ± 0.0452
0.92	0.00105 ± 0.00206	0.000865 ± 0.00104

* *Statistically significantly higher with respect to baseline (p < 0.05) fo, Components' central frequency*.

**Figure 3:**
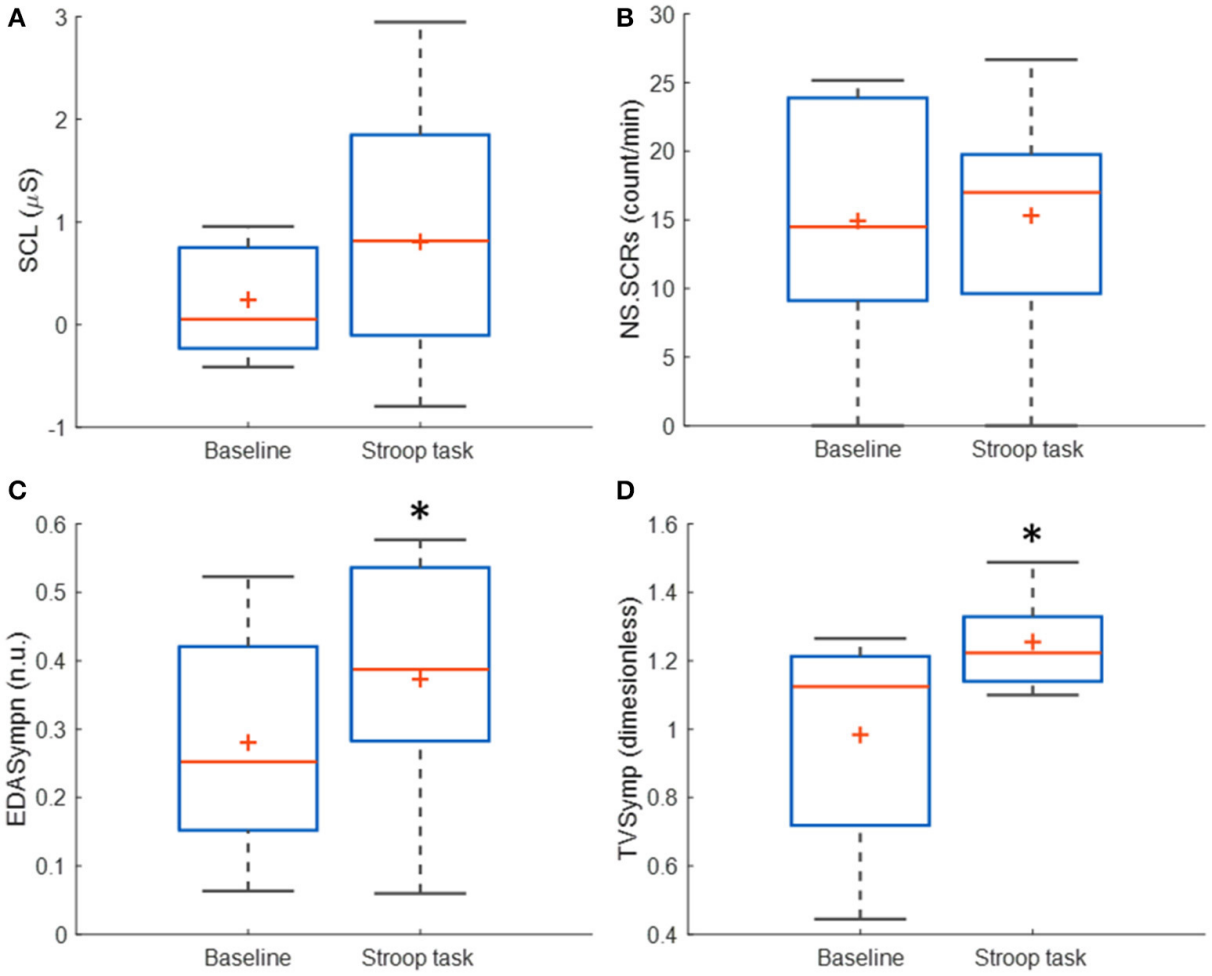
Box plots of the time-domain and frequency-domain measures of EDA for baseline and Stroop task stages, for *N* = 14 subjects immersed in water. (^*^) Represent significant differences between stages. **(A)** SCL, skin conductance level; **(B)** NS.SCRs, non-specific skin conductance responses; **(C)** EDASympn, normalized power spectra in the 0.045 to 0.25 Hz band; **(D)** TVSymp, time-varying index of EDA.

Table 3 incorporates the results of SCL, NS.SCRs, EDASympn and TVSymp for all subjects. The time-domain measures (SCL, NS.SCRs) are not significantly increased during Stroop task, compared to baseline. The tonic component of EDA, which is used to compute the SCL, was highly variable in this study, compared to the mean. EDASympn (normalized power of the 0.045 to 0.25 Hz range) and TVSymp, the spectral-analysis indices of EDA were significantly increased by the Stroop test, compared to baseline stage. Although there was not a significant difference in the amount of phasic reactions observed in the number of NS.SCRs, the EDA was significantly increased in the phasic components assessed quantitatively by the spectral indices. Figure 3 includes box plots for the obtained measures of EDA, for baseline and Stroop task stages.

**Table 3:** Measures of EDA underwater.

**Indices of EDA**	**Baseline**	**Stroop test**	**AUC**	**J**
SCL	0.241 ± 0.545	0.806 ± 1.4	0.67	0.46
NS.SCRs	14.9 ± 9.46	15.3 ± 8.91	0.51	0.23
EDASympn	0.28 ± 0.166	0.373 ± 0.196^*^	0.64	0.38
TVSymp	0.983 ± 0.327	1.25 ± 0.14^*^	0.68	0.42

*Values are expressed as mean ± standard deviation*.

* *Statistically significantly higher with respect to baseline (p < 0.05)*.

*SCL, skin conductance level; NS.SCRs, non-specific skin conductance responses, EDASympn, normalized power spectra in the 0.045 to 0.25 Hz band; TVSymp, time-varying index of EDA; J, Youden's index*.

**Figure 4:**
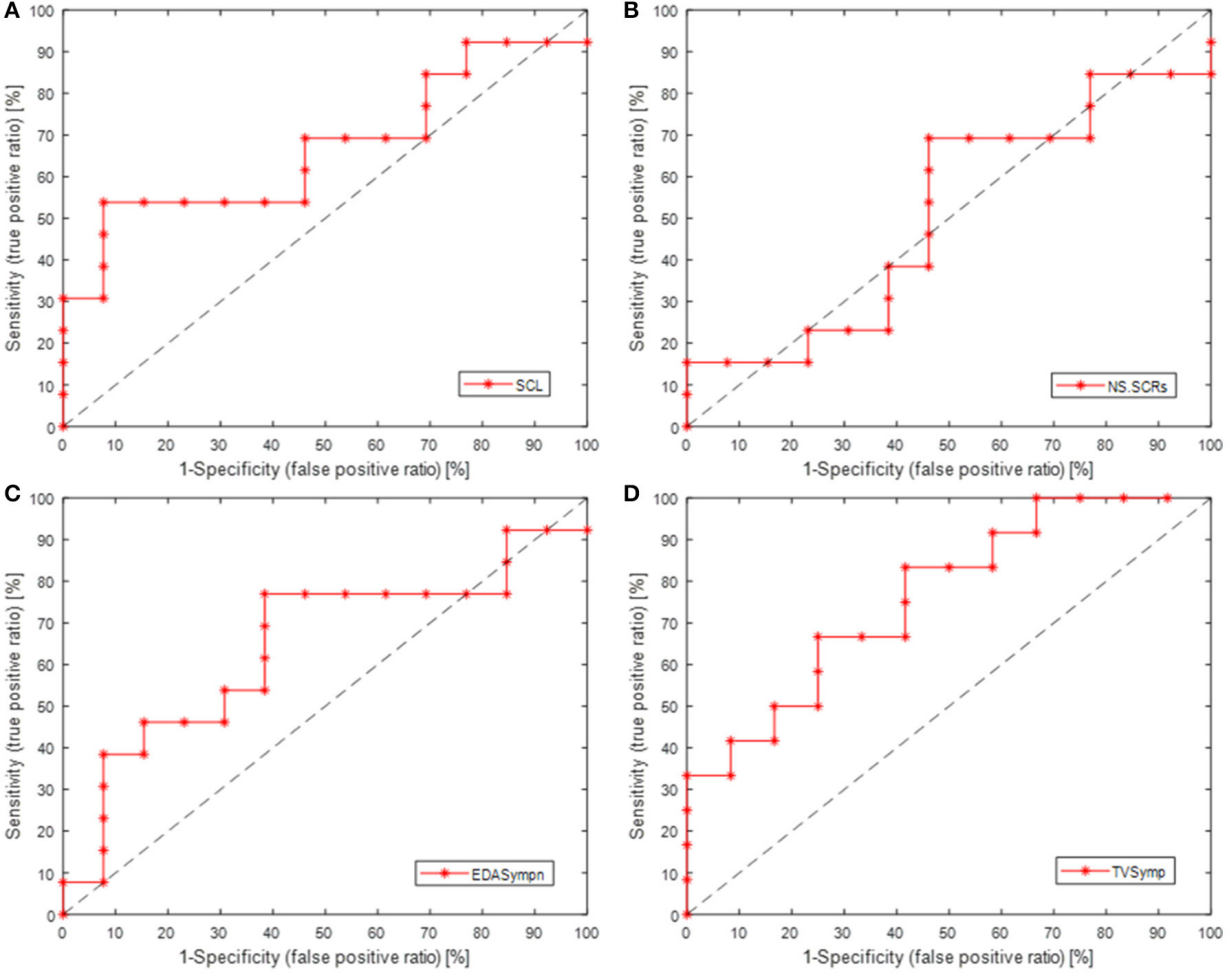
ROC curves (sensitivity vs. 1-specificity) for the measures of EDA, as detectors of cognitive stress induced by Stroop task underwater. **(A)** SCL, skin conductance level; **(B)** NS.SCRs, non-specific skin conductance responses; **(C)** EDASympn, normalized power spectra in the 0.045 to 0.25 Hz band; **(D)** TVSymp, time-varying index of EDA.

Table 3 also incorporates indices from detection theory analysis, which indicate the sensitivity of the measures of EDA, including the Youden's index (J = Sensitivity + Specificity − 1, an indicator of the performance of the detector) (Youden, 1950), and the area under the receiver operating characteristic (ROC) curve (AUC, the probability that the index will assign to a positive instance a higher value than to a negative one) (Hanley and McNeil, 1982). The ROC curves are show in Figure 4 (Metz, 1978). SCL, TVSymp and EDASymp exhibited better performance as discriminators of cognitive stress induced by the Stroop task, compared to NS.SCRs.

## Discussion

The main goal of this work was to determine if cognitive stress can be detected using EDA when subjects are immersed in water. The time-domain and frequency-domain indices based on analysis of EDA were computed for subjects who underwent Stroop task. Time-domain indices were not sensitive enough to detect indication of cognitive stress under water when compared to no Stroop test baseline values. However, time-invariant and time-varying spectral indices exhibited significant differences during the Stroop task, compared to baseline. In general, we found supporting evidence that the phasic component of EDA, assessed by either time-invariant or time-varying spectral analysis, is sensitive to cognitive stress in subjects immersed in water, and can be used to assess the presence of cognitive stress in divers.

There is an increased interest in EDA as an alternative for assessing sympathetic dynamics because sweat glands are only innervated by sympathetic nerves. Using time-invariant spectral analysis of EDA data, we previously found that the band 0.045 to 0.25 Hz is the most sensitive to cognitive, postural and physical stress (Posada-Quintero et al., 2016a). Although sweat glands (sympathetic-cholinergic system) were initially thought to respond only to peripheral stimulus (i.e., thermoregulatory sweating), the electrodermal response is inhibited in response to pharmacological central depressants in a manner analogous to its action on other sympathetic systems (Koss and Davison, 1976b; Girardot and Koss, 1984). This suggests that a central adrenergic inhibitory mechanism is also involved in the regulation of the EDA (Koss and Davison, 1976b; Shields et al., 1987). As EDA can be used as a model system to study the effect on central structures in dry conditions (Koss and Davison, 1976a), we surmise it can be also used to assess the level of stress faced by humans in immersed conditions. To our best knowledge, this has not been tested before.

The high stress observed in a group of subjects, which concealed the Stroop task effects, suggests that to detect changes in EDA due to cognitive stress, subjects need to be trained to be tolerant to water immersion. Otherwise, EDA will be responsive to stress, but the stress caused by immersion will be too high, impeding other influences one is trying to observe. Although the first 2 min were intended as a relaxing baseline measurement underwater, immersion conditions induced increasing stress to subjects. Breathing through the mouth was reported to be stressful, as this maneuver is not the natural way of breathing.

Breathing can also explain the high variability of the tonic (level) changes observed in the EDA in subjects immersed in water (Table 3). Underwater breathing is controlled by the subjects in a different manner than outside the water. Breaths are generally deeper. Deep respiration is known to elicit a generally phasic sympathetic discharge, causing a small increase in sweating, which increases electrical conductivity of the skin (Boucsein et al., 2012). The deep breath is used in practice as a test of subjects' EDA responsiveness. Another possible explanation for the tonic-component variability is the high humidity present in the environment. As the water was warm, and constantly releasing steam, the skin could be constantly collecting water from the environment. Although we tried to perform baseline measurements for more than 20 min, the tonic drift was never stabilized.

A different phenomenon can be observed in the phasic component of EDA, which NS.SCRs, EDASymp and TVSymp account for (Table 3). Although SCRs are not increased in frequency of occurrence, they are increasing in amplitude. That is the reason why the NS.SCRs index is not sensitive to cognitive stress in this study, as for this index a threshold is fixed and SCRs reaching such threshold are considered. On the other hand, EDASymp and TVSymp account for the amplitude of the phasic components associated with the SCRs. As detectors of cognitive stress, EDASymp and TVSymp exhibited acceptable performance, as shown in Figure 4 and Table 3. SCL achieved better performance overall, because apparently this index had a higher probability to assign a higher value to the cognitive stress stage than to the baseline stage. As mentioned before, usually SCL constantly increases due to surrounding humidity. The reason for SCL not to achieve statistical significance between baseline and Stroop task was its high variability.

In this very first exploration of EDA response underwater, the harsh condition of having the electrodes under the diving suit, which will put them in constant friction with subjects' diving suits and introduce motion artifacts (Taamneh et al., 2017), and other circumstances that are known to impair the quality of EDA signals (Boucsein et al., 2012), were not considered, nor the signal processing needed to overcome such signal contamination. Further research needs to be done to examine these effects, which were not the purpose of the current work. In addition, Stroop task was completed by subjects wearing a snorkel and mentally noting the name of the color, which might have limited the effectiveness of inducing cognitive stress to the subjects.

While the number of subjects enrolled for this study was relatively low, significant differences between baseline and stimuli-induced conditions enabled the opportunity to examine if time- and frequency-domain indices can discriminate between the absence and presence of cognitive stress, in water-immersed subjects. In summary, EDA is significantly modified by cognitive stress underwater, which means that EDA is mainly responding to central mechanisms.

## Author contributions

HP-Q and KC: conceived and designed the experiments; HP-Q: carried out the experiments and processed the data. KC, HP-Q, AO-C, and JF: contributed to the analysis of the results. HP-Q and KC: wrote the paper.

### Conflict of interest statement

The authors declare that the research was conducted in the absence of any commercial or financial relationships that could be construed as a potential conflict of interest.
